# mRNA Decay Mediates Evolution and Biological Functions in Plant Gene Expression

**DOI:** 10.1111/pbi.70784

**Published:** 2026-11-01

**Authors:** Haidan Wu, Dayin Lv, Yelin Huang, Yuchi He

**Affiliations:** ^1^ School of Life Sciences Hubei University Wuhan China; ^2^ State Key Laboratory of Biocontrol and Guangdong Provincial Key Laboratory of Plant Stress Biology, Innovation Center for Evolutionary Synthetic Biology, School of Life Sciences Sun Yat‐sen University Guangzhou China

**Keywords:** crop improvement, LECA complexity, mRNA decay, mRNA stability, plant development, stress adaptation

## Abstract

mRNA decay is a key post‐transcriptional regulatory mechanism that fine‐tunes plant development and stress adaptation through dynamic mRNA stability control, bridging fundamental biology and crop improvement. Current knowledge of mRNA decay mechanisms in plants, largely derived from *Arabidopsis*, is poorly generalisable across plant lineages owing to insufficient exploration of other species. Recent advances in high‐throughput sequencing have accelerated the discovery of mRNA decay mechanisms. Here, we systematically illustrate its core molecular mechanisms alongside breakthrough advances. We further dissect evolutionary characteristics of highly conserved core components and lineage‐specific adaptive variations across eukaryotes. Notably, conserved since the last eukaryotic common ancestor (LECA), this machinery reflects a complex ancestral eukaryote and provides high‐resolution phylogenetic markers for reconstructing eukaryotic origins. We also clarify the essential biological roles of mRNA decay in developmental modulation and stress responses. Finally, we propose precision breeding strategies based on mRNA stability regulation to develop high‐yielding, climate‐resilient crops. This review integrates up‐to‐date mechanistic evidence and comparative evolutionary perspectives, consolidates consensus findings and sorts out inconsistent research outcomes, and seeks to enrich the fundamental biological understanding of plant mRNA stability modulation.

## 1 Introduction

Gene expression has long been regarded as primarily regulated at the transcriptional level. However, accumulating evidence now demonstrates that mRNA decay is an essential post‐transcriptional process within the gene regulatory network and is deeply rooted in fundamental plant biology (Dowdle and Lykke‐Andersen 2025; Floris et al. 2009; Qi et al. 2026; Rambout and Maquat 2024; Zhong et al. 2023). Notably, with the continuous maturation of multi‐omics technologies and functional validation systems in recent years, plant mRNA decay research remains in a stage of rapid development. Novel regulatory pathways and evolutionary patterns are constantly emerging, while existing knowledge is being updated and refined.

In plants, accumulating studies have established that mRNA decay is deeply integrated into the precise regulation of plant physiology. By dynamically controlling mRNA stability, it ensures fidelity in genetic information flow, drives rapid transcriptome remodelling, and efficiently degrades aberrant transcripts (Belasco 2010; Deng et al. 2023; Huang et al. 2025; Li et al. 2023; Rambout and Maquat 2024; Wu, Yu, et al. 2024). As sessile organisms, plants cannot actively avoid adverse environmental conditions, which necessitates rapid and flexible reprogramming of gene expression (Jung et al. 2020; Nguyen et al. 2015; Zhu 2016). This inherent constraint renders mRNA decay essential for mediating developmental plasticity, stress responsiveness, environmental adaptation, and cell fate specification (Chantarachot and Bailey‐Serres 2018; Garcia et al. 2014; Huang et al. 2025; Jung et al. 2020; Li et al. 2023; Luha et al. 2024; Napier et al. 2023; Nguyen et al. 2015; Rambout and Maquat 2024; Smith et al. 2024; Yan et al. 2025). For instance, in *Arabidopsis thaliana* , the m^6^A reader proteins ECT2, ECT3 and ECT4 specifically stabilize m^6^A‐modified transcripts associated with ABA signalling via interaction with PAB2 and PAB4; loss of these readers triggers degradation of target transcripts, causes aberrant *ABI5* accumulation, and confers ABA hypersensitivity, thereby modulating seed germination and seedling growth (Song et al. 2023). In rice ( *Oryza sativa* ), *EOG1* shapes grain size by stabilizing mRNAs encoding key regulators of spikelet hull development, providing a potential target for cross‐crop yield improvement (Yan et al. 2024).

However, research on plant mRNA decay has mainly focused on *Arabidopsis*, whereas systematic studies in other plants, such as major cereal crops like rice, wheat, and maize, remain limited (Li et al. 2018; Ma et al. 2025; Sorenson et al. 2018; Tang et al. 2024; Wu, Yu, et al. 2024; Yan et al. 2024; Zhang and Guo 2017). While the core mechanisms of mRNA decay show evolutionary conservation across plant lineages, they remain incompletely characterized in most species, with their lineage‐specific regulatory modalities largely unexplored (Causier et al. 2017; Dowdle and Lykke‐Andersen 2025). These research biases not only hinder the establishment of conserved cross‐species regulatory principles but also limit our comprehensive understanding of how mRNA decay shapes plant diversity and adaptation. In addition, systematic investigations of plant mRNA decay have lagged somewhat behind those in yeast and mammals. For instance, the three major mRNA quality‐control pathways, namely nonsense‐mediated decay (NMD), non‐stop decay (NSD), and no‐go decay (NGD), were well characterized in yeast and animal systems long before their functional validation in plants (Kastenmayer and Green 2000; Losson and Lacroute 1979; Szádeczky‐Kardoss, Csorba, et al. 2018; Szádeczky‐Kardoss, Gál, et al. 2018; Zhang and Guo 2017). To provide a clear historical context for the following discussion, we summarize landmark advances in this field in a timeline (Figure 1). Against this backdrop, this review integrates current knowledge of plant mRNA stability, systematically discusses unresolved puzzles, and highlights prospective cross‐species research directions.

**FIGURE 1:**
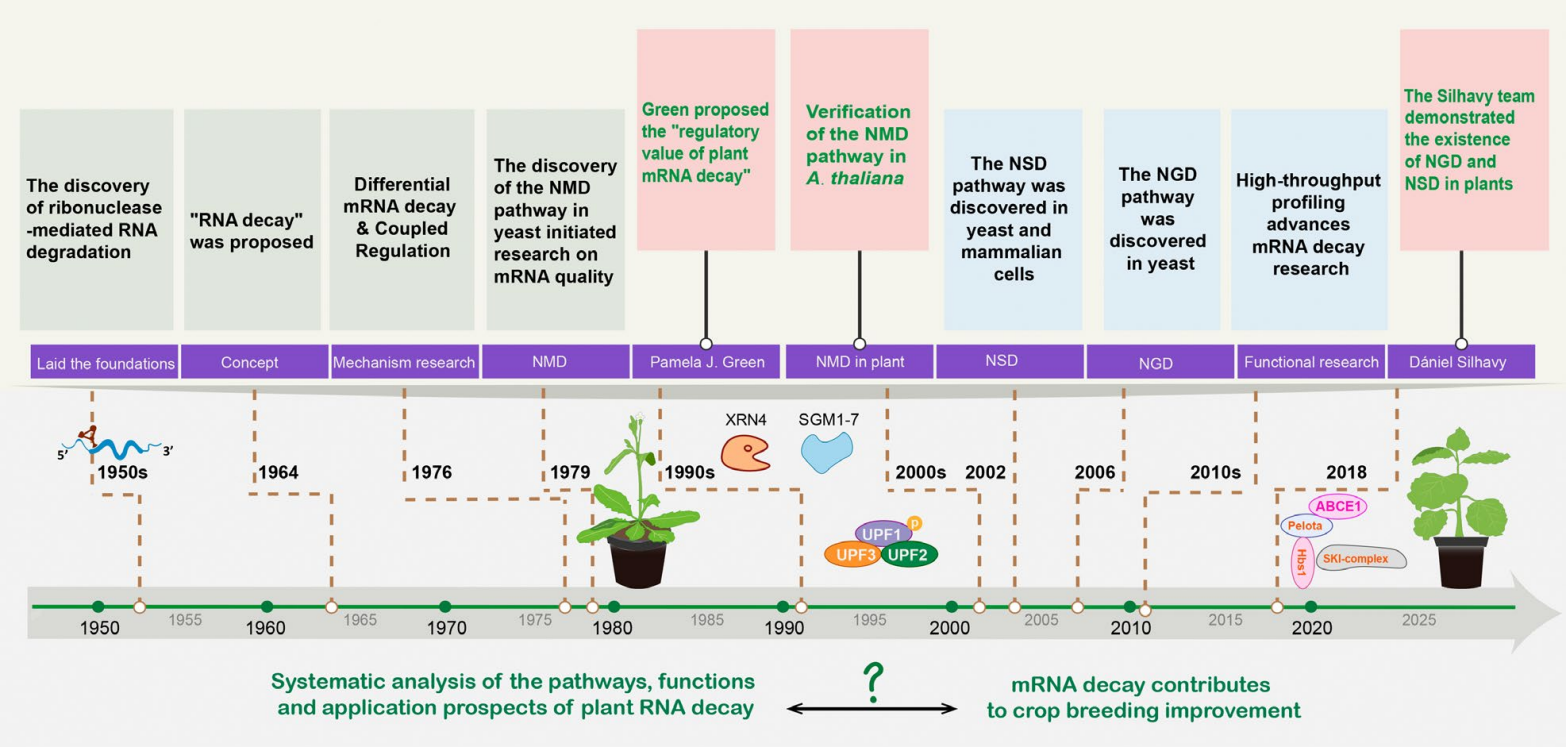
Key milestones in plant mRNA decay research. Early biochemical work from the 1950s to 1970s identified ribonuclease activity, formalized the concept of RNA decay, and uncovered coupled transcription‐translation‐decay crosstalk in yeast and mammals (Cohn and Volkin 1951; Grunberg‐Manago et al. 1955; Markham and Smith 1952; Stiles et al. 1976; Walker et al. 1976). Plant mRNA decay research emerged later; Pamela J. Green's foundational work in the 1990s established mRNA stability as a vital post‐transcriptional regulatory layer (Green 1993). Early 2000s studies in *A. thaliana* delineated plant NMD and confirmed XRN4 as the major cytoplasmic 5′‐3′ exonuclease (Arciga‐Reyes et al. 2006; Kastenmayer and Green 2000; Kertész et al. 2006; Shi et al. 2012; Yoine et al. 2006; Chiba and Green 2009). While NSD and NGD were first characterized in yeast and mammals in the 2000s, the Silhavy lab provided definitive experimental validation of these two surveillance pathways in plants in 2018, uncovering their characteristic substrates and core Pelota–HBS1–SKI2 complexes (Szádeczky‐Kardoss, Csorba, et al. 2018; Szádeczky‐Kardoss, Gál, et al. 2018; Benkovics et al. 2011; Kerényi et al. 2013; Nyikó et al. 2013; Riehs et al. 2008). Subsequent progress includes the characterization of core decay machinery and the rise of high‐throughput technologies, driving research toward future applications in crop breeding.

## 2 Core Molecular Mechanisms in Plant mRNA Decay

Plant mRNA turnover is governed by interconnected decay pathways, spatial compartmentalization, and regulatory determinants, which collectively fine‐tune RNA homeostasis in response to cellular and environmental demands. Notably, while previous reviews have established key foundations for understanding plant mRNA decay, they were constrained by the research progress and scope available at their time of publication. They thus failed to fully introduce recently uncovered mechanistic details (e.g., SUMOylation of PELOTA in NSD) and show a clear bias toward specific model species (Ge et al. 2023). To refine the field's knowledge system, this review comprehensively surveys the literature, integrating classic work and recent breakthroughs to systematically synthesize and clarify the core regulators, specific substrates, molecular mechanisms, multi‐layered regulatory determinants, and spatial‐compartmental controls for plant mRNA decay research.

### 2.1 Deadenylation‐Mediated 5′‐3′ and 3′‐5′ mRNA Decay Pathways

Deadenylation‐mediated mRNA decay is the major pathway, including both 5′–3′ and 3′–5′ mRNA decay (Figure 2a). In plants, the predominant pathway governing cytoplasmic mRNA stability is the deadenylation‐mediated 5′–3′ mRNA decay, whose decapping and subsequent degradation largely take place within processing bodies (P‐bodies) (Matinahmadi et al. 2026; Sorenson et al. 2018; Xu and Chua 2011; Zhang and Guo 2017). Nevertheless, nearly all mechanistic data characterising this core decapping‐dependent decay machinery originate from functional investigations in *A. thaliana* . Resolving this knowledge deficit necessitates cross‐lineage comparative functional investigations in future work.

**FIGURE 2:**
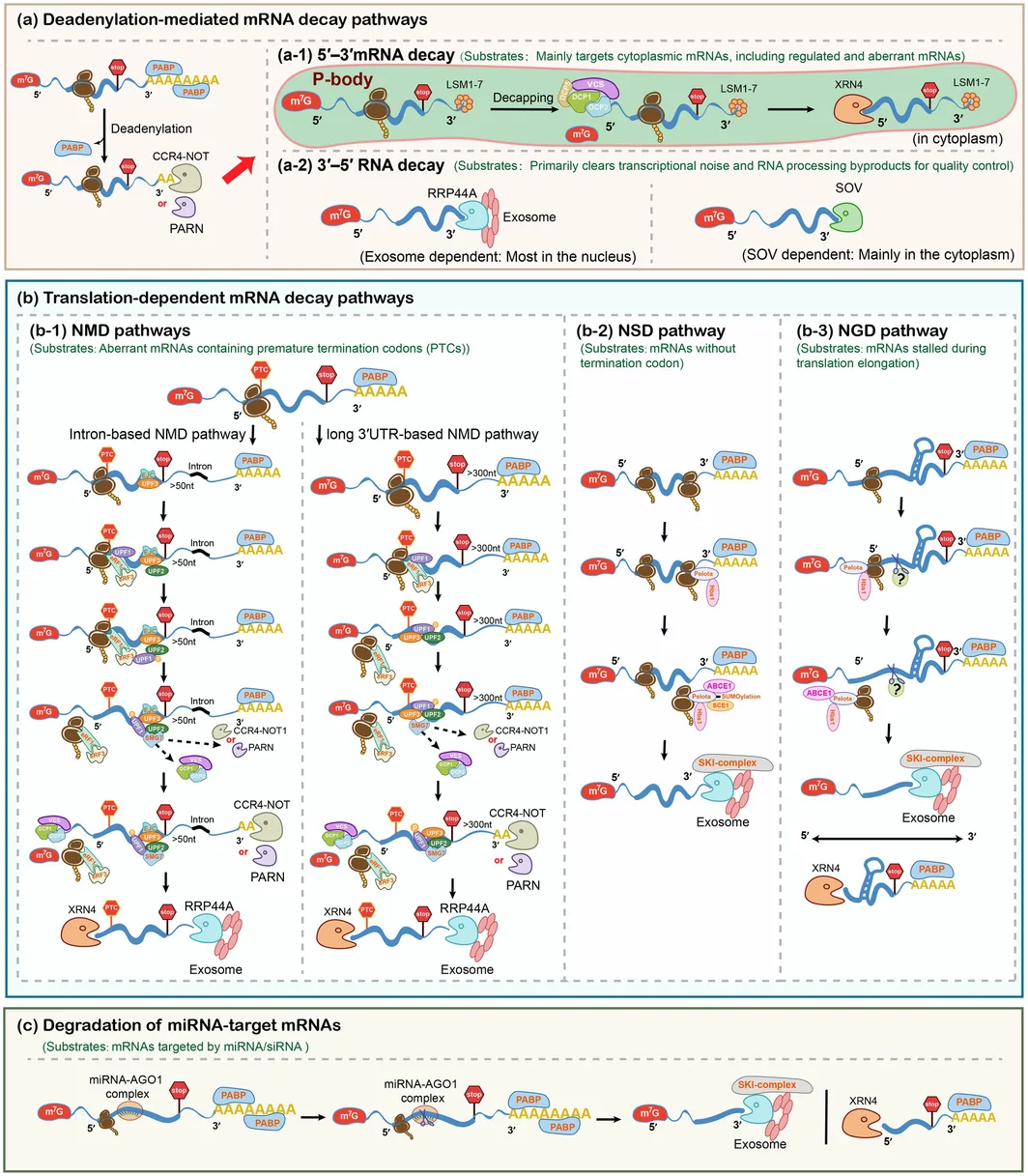
Schematic of multiple mRNA decay pathways in plants. (a) Deadenylation‐mediated mRNA decay pathways; (a‐1) 5′–3′ decay: CCR4–CAF1–NOT1/PARN‐mediated deadenylation → LSM1‐7 recruits VCS‐enhanced DCP1–DCP2 to decap the m^7^G cap → XRN4 degrades cytoplasmic RNAs in P‐bodies. (a‐2) 3′–5′ decay: Deadenylated substrates are mostly degraded by exosome (Exo9 core + RRP44A) in nucleus; SOV/RRP44B acts rarely in cytoplasm. (b) Translation‐dependent mRNA decay pathways, (b‐1) NMD pathways, intron‐based NMD pathway: PTC‐mRNA + intron > 50 nt downstream of stop codon → UPF3‐EJC recruits UPF2, assembling with UPF1 and eRF1/eRF3 into NMD complex → Activated UPF1 recruits SMG7 → exosome (3′‐5′)/XRN4 (5′‐3′) mediate decay; long 3′ UTR‐based NMD pathway: PTC‐mRNA + long 3′ UTR → eRF3 recruits UPF1, followed by UPF2/UPF3 → Activated UPF1 recruits SMG7, leading to mRNA decay via exosome and XRN4. (b‐2) NSD: Non‐stop mRNA triggers ribosome stalling → PELOTA‐HBS1/SKI‐exosome‐mediated 3′‐5′ decay; in *Arabidopsis*, SCE1‐mediated PELOTA SUMOylation is essential. (b‐3) NGD: Ribosome stalling during translation → endonucleolytic cleavage → SKI‐exosome/XRN4 degradation; longer A‐stretches enhance cleavage. (c) Degradation of miRNA‐target mRNAs: AGO1/RISC cleaves target mRNAs → bidirectional degradation by SKI‐exosome/XRN4.

In *Arabidopsis*, 5′–3′ degradation is initiated by deadenylation mediated by the CCR4–NOT complex or PARN, following PABP binding to uridylated oligo(A) tails (Figure 2a‐1) (Reverdatto et al. 2004; Wang, Li, et al. 2024; Zhang and Guo 2017; Zuber et al. 2016). The cytoplasmic LSM1–7 complex recruits the VCS (EDC4)–enhanced DCP1–DCP2 decapping complex to remove the m^7^G cap, producing 5′‐monophosphorylated RNA (Figure 2a‐1) (Golisz et al. 2013; Perea‐Resa et al. 2016, 2012; Sorenson et al. 2018; Xu et al. 2006). Then, cytoplasmic XRN4 degrades decapped transcripts or miRNA‐cleaved products (Figure 2a‐1) (Han, Hou, et al. 2023; Nagarajan et al. 2013). Additionally, the aberrant nuclear mRNAs can be degraded by XRN2/3 (Han, Hou, et al. 2023; Krzyszton et al. 2018; Nagarajan et al. 2013). Notably, functional characterization of the *Arabidopsis* XRN family revealed that XRN4 functionally replaces yeast Xrn1p and thereby acts as the cytoplasmic 5′ → 3′ exonuclease responsible for mRNA degradation (Chiba and Green 2009; Kastenmayer and Green 2000).

Similarly, 3′–5′ mRNA decay also initiates with deadenylation (Figure 2a‐2) (Chiba and Green 2009). RNA substrates are delivered to the exosome complex comprising the nine‐subunit Exo9 core and the catalytic subunit RRP44A, and are degraded 3′→5′ via the central channel (Chekanova et al. 2007; Kumakura et al. 2013; Sikorska et al. 2017; Vuković et al. 2016). This pathway mainly removes nuclear transcriptional noise and RNA processing byproducts, whereas cytoplasmic 3′–5′ mRNA decay is catalysed by SOV/RRP44B in rare instances (Chekanova et al. 2007; Kumakura et al. 2013; Sorenson et al. 2018; Zhang and Guo 2017).

### 2.2 Translation‐Dependent mRNA Quality Control Decay Pathways in Plants

Translation‐dependent mRNA decay pathways serve as mRNA quality control mechanisms in plants, including NMD, NSD, and NGD (Figure 2b). These pathways share the essential function of eliminating aberrant mRNAs to safeguard the fidelity of gene expression (Monaghan et al. 2023; Qi et al. 2026; Zhang and Guo 2017). All three surveillance pathways are triggered during active translation, driven by distinct ribosome stalling or premature termination signals that mark defective transcripts incapable of encoding functional proteins (Monaghan et al. 2023; Zhang and Guo 2017). Among these pathways, NMD was the first identified, and its role in degrading mRNAs containing premature termination codons (PTCs) was established in yeast in 1979 (Losson and Lacroute 1979). Plant NMD was first established in the early 2000s with the characterization of *upf* mutants in *Arabidopsis* (Arciga‐Reyes et al. 2006; Kastenmayer and Green 2000; Kertész et al. 2006; Shi et al. 2012; Yoine et al. 2006). However, NSD and NGD lacked experimental support in plants for a long time until the Silhavy lab validated their existence in 2018 (Szádeczky‐Kardoss, Csorba, et al. 2018; Szádeczky‐Kardoss, Gál, et al. 2018; Zhang and Guo 2017).

Compared with the less‐studied NSD and NGD pathways, NMD is the best‐characterized mRNA quality control pathway in plants. NMD recognizes and degrades mRNAs containing cis‐elements such as PTCs, long 3′ UTRs (> ~300 nt), and introns ≥ 50 nt downstream of the stop codon (Kalyna et al. 2012; Kertész et al. 2006; Nyikó et al. 2013; Shaul 2015). Degradation is activated via either the intron‐dependent pathway (UPF3 binds the EJC to form complexes) or the long 3′ UTR‐dependent pathway (eRF3 recruits UPF1) (Figure 2b‐1) (Dai et al. 2016; Du et al. 2024; Kertész et al. 2006; Luha et al. 2024). NMD is driven by UPF1–3 together with SMG7, the specific SMG‐family effector in plants (SMG5 and SMG6 are absent in plants, and SMG1 is also lacking in *Arabidopsis* and several other species) (Figure 2b‐1) (Luha et al. 2024; Mérai et al. 2013; Shaul 2015).

Unlike the well‐characterised NMD pathway, plant NSD and NGD were uncovered at a later stage. The Silhavy lab functionally defined NSD in *Nicotiana benthamiana* and identified its core Pelota‐HBS1 components, providing critical experimental support for plant mRNA quality‐control networks (Szádeczky‐Kardoss, Csorba, et al. 2018). The substrates of NSD are nonstop mRNAs (mRNAs lacking a termination codon). Upon ribosomal stalling at the mRNA 3′ end, the Pelota‐HBS1 complex can be recruited (Figure 2b‐2) (Szádeczky‐Kardoss, Csorba, et al. 2018). The helicase activity of the SKI complex assists the exosome in binding to the mRNA 3′ end and drives degradation (Szádeczky‐Kardoss, Csorba, et al. 2018; Zhang and Guo 2017). In plants, the plant‐specific SCE1‐mediated SUMOylation of Pelota is critical for complex assembly and the triggering of degradation (Ge et al. 2023).

Meanwhile, the Silhavy lab validated the NGD pathway in *Arabidopsis*, identifying mRNAs with long A‐stretches as its characteristic substrates (Szádeczky‐Kardoss, Gál, et al. 2018). Their work revealed that Pelota–HBS1 is dispensable for NGD‐associated endonucleolytic cleavage but essential for clearing 5′‐cleaved fragments (Figure 2b‐3). Long A‐stretches trigger ribosome stalling, which initiates cleavage by an as‐yet‐unknown endonuclease. Following this cleavage, Pelota–HBS1 binds to the ribosomal empty A site, and ABCE1 facilitates ribosome recycling (Kong et al. 2021; Szádeczky‐Kardoss, Gál, et al. 2018). The resulting mRNA fragments are degraded by the SKI–exosome complex (3′–5′) and XRN4 (5′–3′) (Szádeczky‐Kardoss, Gál, et al. 2018).

### 2.3 Small RNA‐Mediated mRNA Decay Pathways in Plants

Small RNA‐mediated degradation pathways serve as important post‐transcriptional regulatory mechanisms in plant stress responses (Wu et al. 2020; Yan et al. 2025). Following processing by Dicer‐like (DCL) proteins, miRNAs and siRNAs are incorporated into ARGONAUTE1 (AGO1) to form the RNA‐induced silencing complex (RISC), which targets mRNAs through partial complementarity (miRNAs) or perfect base pairing (siRNAs) (Figure 2c) (Branscheid et al. 2015; Wu et al. 2020). mRNA fragments produced by AGO1‐mediated cleavage are subsequently degraded by the SKI–exosome complex and XRN4, although the detailed molecular mechanisms remain unknown (Branscheid et al. 2015).

### 2.4 Multi‐Layered Determinants of Plant mRNA Decay

As summarized above, these canonical mRNA decay pathways fundamentally dictate the specificity and enzymatic mechanisms of mRNA degradation. Nevertheless, identical decay pathways produce markedly different decay rates for distinct cellular transcripts. The stability of these mRNAs is not only dictated by decay pathways themselves, but also fine‐tuned by multiple layers of regulatory factors. Devoid of ribonuclease catalytic activity, these factors shape substrate preference, reaction efficiency and spatiotemporal activity of canonical decay pathways by reshaping molecular interactions, reorganizing subcellular microenvironments and perceiving stress signals (Chantarachot and Bailey‐Serres 2018; Li et al. 2018; Wu, Yu, et al. 2024). Serving as regulatory factors, they dictate whether a given mRNA undergoes degradation, as well as its decay rate.

Intrinsic transcript sequence features may act as kinds of regulatory determinants, including intron presence, sequence length, nucleotide composition, 3′ UTR architecture, codon bias, and specific motifs. For instance, introns can significantly enhance mRNA stability, and the intronless transcripts show faster overall decay compared to intron‐containing ones in *Arabidopsis* and wheat (Narsai et al. 2007; Sorenson et al. 2018; Wu, Yu, et al. 2024). Codon optimality has been established as a key determinant of mRNA stability in yeast, zebrafish, and humans; however, its contribution to mRNA decay is only modest at the genome‐wide scale in wheat (Bazzini et al. 2016; Presnyak et al. 2015; Wu, Yu, et al. 2024). Furthermore, the 3′ UTR may play an important role in determining mRNA decay, harbouring diverse functional sites—polyadenylation signals, stability‐related motifs, and RBP binding sites (Hardy and Balcerowicz 2024; Liu, Wang, Peng, et al. 2025; Wu, Yu, et al. 2024; Zhu et al. 2020). Distinct from linear sequences, RNA secondary structures within the 3′ UTR, such as stability‐associated RNA structural motifs and RNA G‐quadruplexes (rG4s), can reversibly regulate transcript decay rates (Wu, Yu, et al. 2024; Yang et al. 2020, 2022).

Beyond cytoplasmic post‐transcriptional control, mRNA decay also operates via transcription‐RNA decay coupling at the co‐transcriptional level in the nucleus. Nuclear XRN3 co‐transcriptionally eliminates Pol II‐derived aberrant and read‐through pre‐mRNA cleavage remnants during transcription‐termination quality‐control processes (Han, Hou, et al. 2023; Zeng et al. 2024). The nuclear cap‐binding complex (CBC)‐SE may further govern the fate of nascent transcripts. While a competing‐effector‐switch mechanism for routing RNAs toward decay or maturation is structurally supported in humans (Dubiez et al. 2024), in plants, CBC‐SE participates in pri‐miRNA surveillance and cooperates with the NEXT complex for nuclear RNA turnover, although such competitive effector‐driven partitioning remains incompletely validated in planta (Bajczyk et al. 2020). Such nuclear‐resident surveillance establishes regulatory layers prior to mRNA nuclear export.

Notably, RNA modification‐mediated mRNA decay constitutes another dynamically reversible regulatory layer critical for fine‐tuning transcript fate under stress conditions (Cai et al. 2024; Luo et al. 2023). Among these, m^6^A methylation has emerged as a central research focus. m^6^A modification regulates transcript degradation not by directly triggering decay, but by selectively recruiting reader proteins that translate this modification into distinct decay fates (Boo et al. 2022; Cai et al. 2024; Luo et al. 2023; Murakami et al. 2025). Studies in mammalian systems suggest that m^6^A readers can interact with core NMD factors to relay modified‐RNA signals to the nonsense‐mediated decay pathway (Boo et al. 2022). This crosstalk has been validated in plants, m^6^A readers ECT2A/B/C interact with NMD factors UPF3/SMG7 to degrade m^6^A‐modified viral RNAs (He et al. 2024). Beyond NMD, m^6^A modification also crosstalks with other canonical mRNA decay machineries. In mammals, m^6^A reader YTHDF2 recruits the CCR4–NOT deadenylase complex together with PABP to trigger deadenylation and accelerate mRNA turnover (Du et al. 2016). In *Arabidopsis*, however, the m^6^A reader ECT2 directly interacts with PAB2/PAB4 to stabilize m^6^A‐marked transcripts, whereas plant m^6^A readers have not yet been demonstrated to associate with the CCR4–NOT complex (Song et al. 2023). Recently, CDS‐localized m^6^A was shown to trigger ribosome stalling and collisions, initiating co‐translational RNA decay (CTRD) as a pivotal stress‐responsive surveillance mechanism in plants (Zhong et al. 2026). m^6^A deposition in coding regions stalls ribosomal elongation and induces collisions, suggesting that m^6^A may cooperate with NGD‐/NSD‐mediated surveillance pathways to clear aberrant transcripts (Murakami et al. 2025; Zhong et al. 2026; Zhou et al. 2024). Furthermore, the activity of m^6^A modifications and their reader proteins can be dynamically regulated by developmental and stress signals (Cai et al. 2024; Zhou et al. 2024). For instance, m^6^A reader SiYTH1 forms cytoplasmic condensates through liquid–liquid phase separation (LLPS), paradoxically stabilizing drought‐responsive transcripts and consequently enhancing plant stress tolerance (Luo et al. 2023).

### 2.5 Liquid–Liquid Phase Separation‐Driven mRNA Decay in Plants

Beyond the canonical enzymatic cascades of plant mRNA decay outlined above, accumulating evidence highlights an additional critical spatial regulatory layer mediated by LLPS. This reversible biophysical process drives the assembly of membraneless biomolecular condensates such as processing‐bodies (P‐bodies, PBs) and stress granules (SGs), which establish highly dynamic, tunable reaction compartments for mRNA metabolism (Chantarachot and Bailey‐Serres 2018; Fan et al. 2024; Xu et al. 2021). In plant cells, P‐bodies and SGs represent two major membraneless compartments tightly linked to mRNA storage, translational repression, and mRNA decay (Kearly et al. 2024; Matinahmadi et al. 2026; Xu et al. 2021). Although P‐bodies and SGs are functionally distinct, with P‐bodies enriched in mRNA decay factors and SGs primarily engaged in transient mRNA sequestration under stress, they share a subset of protein and RNA components and undergo dynamic material exchange, collectively governing mRNA fate.

In plants, P‐bodies serve as the core membraneless organelles supporting the majority of cytoplasmic deadenylation‐mediated 5′‐3′ mRNA decay (Xu and Chua 2011). Their assembly and maintenance are critically dependent on LLPS driven by multivalent interactions. DCP5 acts as the principal plant‐specific scaffold for P‐bodies in *Arabidopsis*; possessing intrinsically disordered regions (IDRs) that confer phase‐separation capacity, it nucleates the formation of spherical, dynamically exchangeable liquid condensates (Jang et al. 2019; Matinahmadi et al. 2026; Wang, Yang, et al. 2024; Xu and Chua 2009). Under steady‐state conditions, DCP5 localizes predominantly to P‐bodies; however, upon exposure to heat or osmotic stress, DCP5 redistributes and co‐localizes with canonical SG markers, thereby functioning as a shared component of both P‐bodies and SGs (Matinahmadi et al. 2026; Wang, Yang, et al. 2024). The C‐terminal domain of DCP5 mediates its interaction with DCP1 and DCP2, thereby anchoring the intact decapping complex to P‐bodies (Xu and Chua 2009). Cytoplasmic deadenylation mediated by the CCR4–NOT and PARN complexes can occur across the cytoplasm and is not restricted to P‐bodies (Chantarachot and Bailey‐Serres 2018). Following deadenylation, target mRNAs are recruited into P‐bodies, where deadenylated transcripts, the oligo(A)‐binding LSM1‐7 complex, decapping factors (DCP1/2‐VCS), and cytoplasmic XRN4 are spatially concentrated together, enabling efficient local execution of 5′‐3′ mRNA decay (Figure 2a‐1) (Matinahmadi et al. 2026; Tibble et al. 2021; Wang, Yang, et al. 2024; Xu and Chua 2009). By contrast, 3′‐5′ mRNA decay is predominantly nuclear and carried out by the exosome complex; its cytoplasmic activity represents an auxiliary pathway (Sorenson et al. 2018; Zhang and Guo 2017). To date, no solid evidence supports SOV/RRP44B‐dependent 3′‐5′ degradation inside plant P‐bodies, and P‐bodies are primarily associated with the 5′‐3′ decay pathway (Chantarachot and Bailey‐Serres 2018; Zhang and Guo 2017; Matinahmadi et al. 2026). Furthermore, the interplay between LLPS and translation‐dependent surveillance pathways, including NMD, NGD, and NSD, awaits further in‐depth investigation (Matinahmadi et al. 2026).

Collectively, mRNA decay pathways, transcript sequence features, RNA modifications, phase‐separation‐driven compartmentalization, together with developmental and stress signals, cooperatively confer substrate specificity and environmental plasticity on the plant mRNA decay program. Nevertheless, major gaps remain. For instance, direct evidence from in vitro reconstitution and phase‐separation‐deficient mutants regarding the spatiotemporal crosstalk between decay pathways and membraneless condensates remains scarce. Moreover, current mechanistic knowledge of mRNA decay draws heavily on *A. thaliana* ; further investigation of non‐model plants and staple crops is needed to uncover both lineage‐specific diversity and conserved regulatory principles.

## 3 Conserved Cores and Lineage‐Specific Adaptations in Eukaryotic mRNA Decay Mechanisms

The core molecular mechanisms of mRNA decay in plants have been elucidated, and their broad conservation across species raises a fundamental scientific question: what is the evolutionary pattern of this regulatory system in eukaryotes? Addressing this question will enhance our evolutionary understanding of mRNA decay mechanisms and uncover the fundamental role of RNA turnover in eukaryotic gene expression. In recent years, studies have expanded from model plants to diverse eukaryotic lineages. Causier et al. demonstrated that core NMD factors (e.g., UPF1, SMG1) have functionally conserved orthologs in animals, plants, fungi, and protists, indicating that the NMD pathway might originate in LECA (Causier et al. 2017). Although NMD is broadly conserved, most studies have focused on a few key proteins in yeast, model animals, or plants, with little cross‐lineage analysis (Causier et al. 2017; Dowdle and Lykke‐Andersen 2025; Mattout et al. 2020; Sharif and Conti 2013).

LECA likely arose from a symbiotic merger between an Asgard‐related archaeon and an Alphaproteobacterium (mitochondrial ancestor) (Krupovic et al. 2023; Nobs et al. 2022; Spang et al. 2015; Tobiasson et al. 2026; Williams et al. 2020). As the last common ancestor of all extant eukaryotes (Figure 3a), LECA (dated to 1.93–1.84 Ga, later than LUCA) possesses the cellular complexity proposed by the LECA complexity hypothesis, including a nucleus, endomembrane system, mitochondrion, dynamic cytoskeleton, and cell‐cycle control machinery (Mahendrarajah et al. 2023; O'Malley et al. 2019; Raval et al. 2022; Vosseberg et al. 2024, 2021; Zhang et al. 2025). This complexity likely laid the foundation for subsequent eukaryotic diversification. Remarkably, although most evolutionary studies of mRNA decay have focused on individual core proteins in a single pathway (e.g., NMD), cumulative scattered evidence nevertheless suggests an emerging evolutionary pattern: core regulatory modules of mRNA decay are likely widespread and highly conserved across major eukaryotic lineages. Thus, we raise a question: Is the mRNA decay system an intrinsic component of LECA cellular complexity, thereby providing independent evidence for the LECA complexity hypothesis at the gene expression level (Figure 3a)? If so, this system represents an inherent mechanism enabling high‐fidelity and adaptive gene expression in LECA, rather than a later evolutionary innovation. Here, we systematically analysed core mRNA decay factors (including CCR4–NOT, LSM complexes, DCP1/2, exoribonucleases (e.g., RRP44), and key components of the NMD, NSD, and NGD pathways) across diverse eukaryotes (yeast, animals, algae, bryophytes, ferns, gymnosperms, and angiosperms) using genomic databases including Phytozome (https://phytozome‐next.jgi.doe.gov/) and UCSC Genome Browser (http://genome.ucsc.edu/), to trace their evolutionary origins and try to define the unique features of plant mRNA decay mechanisms.

**FIGURE 3:**
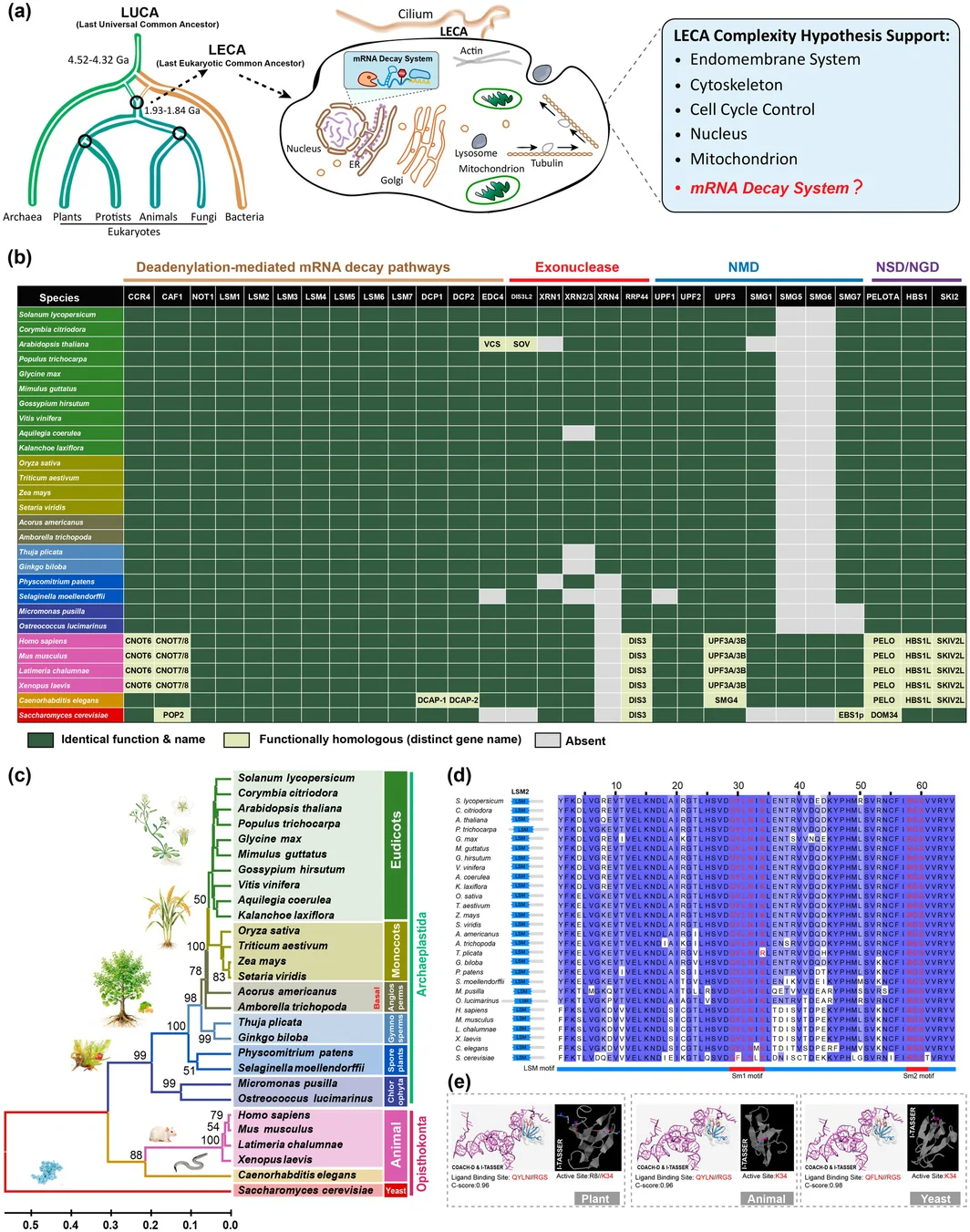
Evolutionary conservation and lineage‐specific differentiation characteristics of mRNA decay machinery across eukaryotes. (a) Schematic overview of eukaryote origin, the LECA complexity hypothesis (LECA was a sophisticated cell equipped with endomembrane system, cytoskeleton, cell cycle regulation system, nucleus and mitochondrion), and the key scientific question raised: Whether mRNA decay system originated in LECA and provided independent evidence for the hypothesis. (b) Systematic profiling of key mRNA decay pathway components across representative eukaryotes, with colour coding indicating homology relationships: Dark green for orthologs with identical function and name, light green for orthologs with functional conservation but distinct names, and grey for absent or significant sequence divergence beyond recognition. (c) Maximum‐likelihood (ML) phylogenetic tree inferred from concatenated LSM1‐7 subunit protein sequences using MEGA. Statistical confidence values on major branches are derived from 1000 bootstrap replications, and the scale bar indicates substitutions per branch length. (d) Cross‐species multiple sequence alignment of the LSM motif from LSM2. The Sm1/2 motif regions are highlighted in red and exhibit near‐identical sequence conservation across diverse eukaryotic lineages. (e) Structural prediction of LSM motif from LSM2 using COACH‐D and I‐TASSER (*C*‐score = 0.96 for plant and animal; *C*‐score = 0.98 for yeast) (Wu et al. 2018; Yang et al. 2015). The 3D architectures of ligand‐binding sites and active sites are nearly identical across plant, animal, and yeast lineages.

### 3.1 Deeply Conserved Core Orthologs in Eukaryotic mRNA Decay

Our analyses reveal that core components of mRNA decay pathways are deeply conserved across major eukaryotic supergroups, inherited vertically from LECA. The phenomenon not only supports the “LECA complexity hypothesis” at the level of gene expression but also underscores the pathway's fundamental role in eukaryotic cell biology (Figure 3b). Taking 5′ → 3′ exoribonucleases as an example, XRN1 acts as the major exonuclease in most eukaryotes, but is replaced by XRN4 in *A. thaliana* and *Physcomitrium patens*. XRN4 is absent in non‐plant lineages, suggesting that plants have reshaped this pathway via functional protein replacement (Chiba and Green 2009; Kastenmayer and Green 2000). Similarly, the NMD pathway retains the core UPF1–3 module across eukaryotes but shows striking lineage‐specific diversification in regulatory kinases: SMG5/6 are found only in animals; SMG1 is absent in *Arabidopsis* and yeast, where its function is compensated by SMG7 (Ebs1p in yeast). By contrast, both SMG1 and SMG7 coexist in rice, maize, and other plants, with their functional divergence yet to be elucidated (Benkovics et al. 2011; Grimson et al. 2004; Kerényi et al. 2013; Luke et al. 2007; Nyikó et al. 2013; Riehs et al. 2008). Remarkably, core regulators of mRNA decay pathways are highly conserved orthologs across eukaryotes, with conserved sequences and functions. They differ in name only due to independent early studies in distinct lineages. For instance, RRP44 in Archaeplastida is known as DIS3 in Opisthokonta, both being orthologs derived from the same ancestral gene (Figure 3b,c).

### 3.2 mRNA Decay Orthologs Serve as Molecular Markers for Deep Eukaryotic Phylogeny

Orthologous mRNA decay proteins with conserved sequences and functions hold great potential in systems biology and evolutionary studies, serving as reliable markers for deep phylogenetic inference across eukaryotes. In this review, we selected the seven protein subunit sequences of the LSM1–7 complex (a conserved heptameric ring complex across eukaryotes) as representative markers (Figure 3c) (Montemayor et al. 2020; Perea‐Resa et al. 2016). Maximum‐likelihood phylogenies inferred from concatenated sequences of the seven LSM1–7 subunits robustly resolve the relationships among major eukaryotic supergroups, including Opisthokonta and Archaeplastida (Figure 3c). To further explore the structural basis of this conservation at the molecular level, we performed multi‐species sequence alignment of the LSM motif region in LSM2. While modest divergence occurs across lineages, the region is highly conserved, particularly the key amino acid residues forming the Sm motif (Figure 3d). Furthermore, protein structure prediction shows that key RNA‐binding residues (e.g., QYLN, RGS, K34) and complex assembly interfaces are strictly conserved across species. Despite modest sequence divergence among lineages, the predicted three‐dimensional structures are strikingly similar across eukaryotic supergroups (Figure 3e).

In summary, core mRNA decay components are deeply conserved across eukaryotes, indicating their origin in LECA and maintenance via vertical inheritance. This finding provides critical additional support for the complex LECA hypothesis. Specifically, the pathway comprises a conserved core scaffold and flexible regulatory elements: the core sustains fundamental function stability, while variable modules evolve adaptively under natural selection. Consistent with recent research, eukaryotes maintain stable core functions while flexibly adjusting regulatory modules to adapt to environmental constraints (Large et al. 2025). Therefore, the core components provide high‐confidence evidence for reconstructing the post‐transcriptional regulatory mechanisms of LECA, as well as reliable molecular markers for eukaryotic phylogenetics. These components are highly conserved with clear orthologous relationships, representing functionally well‐defined post‐transcriptional modules that originated in LECA. Compared with 1:1 ortholog sets in angiosperms or traditional single‐gene markers such as ATPase, multi‐subunit complexes (e.g., LSM1–7, CCR4–NOT, UPF1–3) yield stronger co‐evolutionary markers, improving the robustness and resolution of phylogenetic reconstruction while reducing stochastic errors, horizontal gene transfer, and systematic biases (Mahendrarajah et al. 2023; Schuster et al. 2026). Viewed from systematic biology and eukaryotic evolution, these orthologs surpass their cellular roles and offer key molecular clues to eukaryotic origin, diversification and adaptation. While core decay factors share conserved sequences and structures suggestive of universal eukaryotic mechanisms rooted in LECA, functional verification remains confined chiefly to *Arabidopsis* and limited crop species. It still lacks definitive proof that conserved molecular architecture translates into identical regulatory logic across diverse non‐model organisms. Notably, structural homology does not guarantee uniform functional operation, given the inherent plasticity of regulatory targets alongside stable core machinery.

## 4 Functions of mRNA Decay in Plant Development and Stress Responses

Gene expression modulation underpins plant growth and environmental acclimation. Whereas transcriptional regulation is well‐characterized, dynamic post‐transcriptional control is attracting increasing research attention. As a core post‐transcriptional module, mRNA decay shapes both transcript abundance and translational efficiency; co‐translational mRNA decay, for instance, remodels protein output independently of bulk mRNA levels (Carpentier et al. 2020, 2024; Deragon and Merret 2025; Hou et al. 2022; Radhakrishnan and Green 2016; Smith et al. 2024). By fine‐tuning steady‐state transcript pools and eliminating aberrant mRNAs, mRNA decay governs the timing and amplitude of gene expression (Faucillion et al. 2022; Rambout and Maquat 2024; Timmers and Tora 2018; Wu, Yu, et al. 2024). It contributes to basic metabolism and development and integrates environmental and stress signals to enable dynamic adaptation in plants (Chantarachot and Bailey‐Serres 2018; Chantarachot et al. 2020; Deragon and Merret 2025; Matsui et al. 2019). Recent studies have demonstrated that mRNA decay plays crucial roles in plant development, including seed germination, photosynthesis, and flowering time; organogenesis, such as pollen development, root growth, and leaf morphogenesis; and responses to abiotic stresses (temperature, salt, drought, phosphate deficiency) and biotic stresses (viral, fungal, and insect infection) (Figure 4) (Jiang et al. 2020; Jing et al. 2025; Liu, Wang, Wang, et al. 2025; Liu et al. 2024; Ma et al. 2025; Nasim et al. 2021; Tang et al. 2024; Wang et al. 2023, 2022; Wawer et al. 2018; Yan et al. 2025; Yang et al. 2022).

**FIGURE 4:**
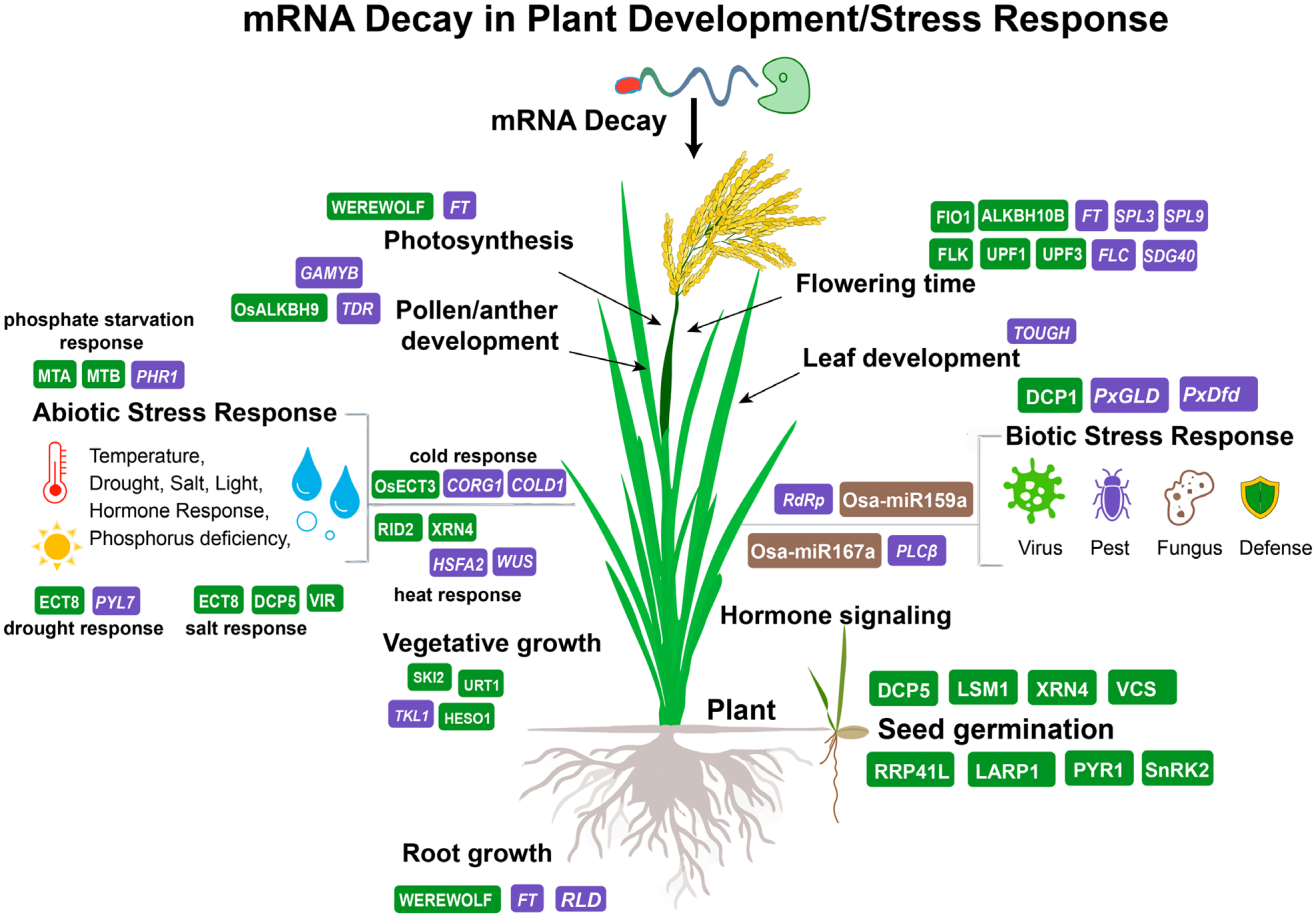
Schematic model of multilayered mRNA decay‐mediated regulation of plant growth and development. Green boxes denote protein regulators of mRNA stability, purple boxes represent their downstream target transcripts, and brown boxes mark miRNAs that direct sequence‐specific transcript cleavage and degradation. Through these diverse yet interconnected pathways, mRNA decay orchestrates diverse developmental programmes—from photosynthesis and vegetative growth to abiotic/biotic stress response—enabling plants to precisely fine‐tune growth and adapt to fluctuating environments.

### 4.1 Dynamic Control of Plant Development Through mRNA Stability Regulation

By fine‐tuning mRNA stability of core developmental regulators, mRNA decay modulates plant life‐cycle progression from seed germination to reproductive growth. In *Arabidopsis*, multiple mRNA decay‐related factors including DCP5, LSM1, XRN4, VCS, RRP41L, and LARP1 are documented to modulate seed germination and early seedling establishment through altering the stability of cytoplasmic transcripts associated with seed storage protein metabolism as well as ABA and GA biosynthetic pathways (Basbouss‐Serhal et al. 2017; Wawer et al. 2018; Yan et al. 2023; Yang et al. 2013). For example, the decapping factors DCP5 and LSM1 modulate ABA signalling via controlling mRNA stability of ABA receptor PYR1 and SnRK2; genetic loss of DCP5 substantially elevates plant ABA sensitivity and severely suppresses seed germination efficiency (Wawer et al. 2018). Beyond seed and seedling‐level responses, mRNA decay also executes cell‐type‐specific control during organ morphogenesis and cell‐fate specification. Current studies on such regulatory events largely focus on two mechanistic routes: sRNA‐guided transcript cleavage and RNA modification‐mediated control of mRNA stability. In grass anthers, 21‐nt phasiRNAs regulate male fertility through AGO‐mediated cleavage and exhibit cell‐type‐specific 5′‐nucleotide preference, with a strong cytosine bias in male germ cells but not in somatic cells, indicating unique RNA regulatory programmes in distinct cell types (Jiang et al. 2020). Another example in rice is that m^6^A‐eraser OsALKBH9 stabilizes *TDR* and *GAMYB* transcripts through m^6^A demethylation, consequently governing tapetum programmed cell death and pollen exine formation for male reproductive development (Tang et al. 2024). Beyond reproductive organs, mRNA decay, often coordinated with RNA modifications, also participates in the regulation of vegetative organ development. In leaf morphogenesis, reduced ac^4^C modification of *TOUGH* (a key gene involved in miRNA biogenesis) accelerates its transcript degradation, disrupts miRNA homeostasis, and ultimately impairs normal leaf development (Wang et al. 2023). In roots, the miR165/166–HD‐ZIP III module regulates vascular tissue differentiation by cleaving target mRNAs such as *RLD*, while gain‐of‐function mutations escape such degradation, resulting in shorter primary roots, fewer vascular cells, and impaired root development (Di Ruocco et al. 2018; Gautam et al. 2021).

### 4.2 Regulation of Physiological Traits by mRNA Decay

Apart from developmental growth regulation, functional crosstalk between mRNA decay and primary metabolic processes has also been gradually revealed. A landmark study clarified that RNA uridyltransferases HESO1 and URT1, together with SKI2, collectively mediate mRNA quality control of *TKL1*, a key Calvin‐cycle gene in *Arabidopsis* (Wang et al. 2022). By degrading aberrant *TKL1* transcripts, they prevent the production of abnormal siRNAs, thereby safeguarding normal photosynthesis (Wang et al. 2022). This work bridges mRNA turnover machinery and core carbon metabolism, underscoring its indispensable role in sustaining intracellular physiological homeostasis. Nevertheless, such crosstalk between mRNA decay and carbon metabolism remains poorly understood; how mRNA decay modulates other key enzymes and pathways of primary carbon metabolism (e.g., glycolysis, tricarboxylic acid cycle) remains largely unexplored, calling for further multi‐omics and interaction assays investigations.

Similar knowledge gaps persist for flowering‐time control, another core physiological trait shaped by mRNA decay machinery. This machinery reshapes the transcript abundance of flowering‐related genes through multiple pathways, including nonsense‐mediated decay (NMD) surveillance, m^6^A modification and miRNA‐mediated regulation (Duan et al. 2017; Nasim et al. 2021). Loss‐of‐function mutations in core NMD components UPF1 and UPF3 lead to massive accumulation of *FLC* transcripts and consequent delayed flowering phenotype (Nasim et al. 2021). Mechanistically, NMD modulates chromatin modification at the *FLC* locus by targeting *SDG40* for degradation, balancing H3K4me3 and H3K27me3 epigenetic marks; consistently, *sdg40* mutants flower early and rescue the late‐flowering phenotype of *upf* mutants (Nasim et al. 2021). Deficiency of the m^6^A eraser ALKBH10B leads to excessive m^6^A deposition and accelerated turnover of florigen‐encoding *FT*, *SPL3* and *SPL9*, resulting in delayed flowering (Duan et al. 2017). Beyond a few m^6^A erasers, m^6^A methyltransferase FIO1 and m^6^A reader FLK also fine‐tune floral transition by governing splicing and stability of *FLC* and other flowering‐associated transcripts (Amara et al. 2023; Cai et al. 2023; Xu et al. 2022). In addition, the root development regulator WEREWOLF participates in non‐cell‐autonomous regulation of *FT* mRNA stability, linking root morphogenesis to photoperiod‐dependent flowering (Seo et al. 2011). However, the molecular mechanisms underlying their synergistic or antagonistic crosstalk in fine‐tuning flowering time remain unclear. For example, how NMD and m^6^A‐related effectors synergistically or antagonistically regulate common targets such as *FT* and *FLC* under fluctuating environments; and whether mRNA decay acts uniformly or adopts cell type specific patterns within floral meristems to establish precise flowering outputs.

### 4.3 mRNA Decay in Plant Abiotic Stress Responses

Plants deploy multiple mRNA decay pathways to perceive and adapt to diverse abiotic cues, with epitranscriptomic marks, especially m^6^A, frequently acting as central molecular switches that rewire transcript turnover under stress. Temperature fluctuation is one major environmental constraint shaping plant performance. In rice, cold‐triggered deacetylation of the m^6^A‐reader OsECT3 at K471 strengthens its binding to m^6^A‐tagged cold‐responsive transcripts such as *COLD1*, stabilizing these mRNAs and boosting cold tolerance (Ma et al. 2025). This finding defines a regulatory cascade connecting lysine acetylation, m^6^A modification and mRNA stability during cold acclimation. In *Arabidopsis*, low‐temperature‐induced formation of RNA G‐quadruplex structures in guanine‐rich transcripts shields cold adaptive mRNAs including *CORG1* from degradation, conferring enhanced cold resistance and revealing an additional mechanism underlying plant cold adaptation (Yang et al. 2022). Similarly, mRNA decay pathways play a crucial role in mediating heat stress responses. The m^7^G methyltransferase RID2 maintains *WUS* mRNA stability and regulates stem cell activity in *Arabidopsis* shoot apical meristems, and high temperature represses RID2 to promote *WUS* mRNA degradation and enhance stem cell thermotolerance (Liu et al. 2024). Under heat stress, AGO1 undergoes LLPS, driving its relocalization into stress‐granule condensates (Blagojevic et al. 2024). Although short‐term heat exerts only modest effects on AGO1‐loaded small‐RNA populations and target‐cleavage signatures, AGO1 interactomes under heat stress recover multiple RNA‐decay components including DCP5 and the NMD core factor UPF1. Overall, these observations raise the prospect of functional crosstalk among phase separation, RNA modification, and canonical mRNA‐decay pathways during heat‐stress adaptation.

Furthermore, drought and salinity represent the other major abiotic environmental constraints that negatively affect plant growth and yield, with m^6^A‐mediated mRNA decay contributing to stress adaptation (Cai et al. 2024; Hou et al. 2022; Wu, Su, et al. 2024). Under drought, the m^6^A demethylase ALKBH10B also modulates drought responses by shaping the stability of drought‐responsive transcripts (Han, Shoaib, et al. 2023). ABA induces the m^6^A reader protein ECT8 to repress *PYL7* translation, and its knockout increases *PYL7* mRNA stability and overactivates ABA signalling, leading to decreased drought tolerance (Wu, Su, et al. 2024). During salt stress, ECT8 recognizes m^6^A‐modified mRNAs and cooperates with decapping factor DCP5 within P‐bodies to accelerate target decay, thereby enhancing salt tolerance (Cai et al. 2024). ECT8 exhibits distinct regulatory patterns across osmotic stresses, and its functional switching mechanism remains unclarified. In addition, the m^6^A writer component VIR enhances salt tolerance by depositing m^6^A in 3′ UTRs to trigger alternative polyadenylation‐dependent 3′ UTR lengthening, which accelerates the decay of mRNAs encoding salt stress negative regulators (Hu et al. 2021). Moreover, under phosphate starvation, the *Arabidopsis* m^6^A writers MTA and MTB elevate transcript stability of key transcription factors including *PHR1* via m^6^A deposition to activate phosphate starvation responses (Liu, Wang, Wang, et al. 2025). Compared with temperature and osmotic stresses, nutrient‐stress‐associated mRNA decay regulation is still at an early research stage and deserves further investigation. Overall, these examples illustrate that m^6^A and associated mRNA decay effectors interpret distinct stress signals by differentially affecting stress‐relevant transcripts.

### 4.4 mRNA Decay Functions in Plant Biotic Stress Responses

mRNA decay machinery constitutes a multi‐layered regulatory hub shaping plant biotic‐stress adaptation, governing plant‐pathogen and plant‐insect interactions and immune output. The research has emerged in recent years, shedding new light on the complexity of mRNA decay‐mediated plant biotic stress responses. The dynamic ‘molecular arms race’ between plants and viruses, centred on mRNA decay pathways, has been well characterized. Host RNA quality‐control pathways including NMD and NGD serve as primary antiviral surveillance layers that recognize and eliminate viral RNAs (Chen, Jia, et al. 2024; Du et al. 2024; May and Simon 2021). In response, viruses have also evolved counter‐defence mechanisms, underscoring the coevolutionary dynamics between hosts and pathogens (Du et al. 2024; Wu et al. 2025). For instance, the turnip crinkle virus capsid protein (CP) mediates AtDcp1 ubiquitination and degradation, thereby suppressing antiviral mRNA decay and promoting viral accumulation (Wu et al. 2025).

Beyond host‐virus arms‐race, epitranscriptomic modifications cooperate with UPF‐dependent NMD to reshape immune‐transcript turnover during bacterial infection. Upon pathogen challenge, m^6^A marks dynamically reprogram the fate of defence‐related transcripts: the m^6^A writer complex targets immune‐induced mRNAs for accelerated turnover, whereas ECT reader proteins enhance their translation, thereby orchestrating a transient yet effective defence surge (Chen, Greene, et al. 2024; Prall et al. 2023). The NMD pathway, represented by core UPF and SMG7 factors, mediates basal turnover of NLR immune‐receptor transcripts (Gloggnitzer et al. 2014; Jung et al. 2020). During bacterial infection, plants attenuate NMD activity to stabilize these defence mRNAs, and NMD‐deficient mutants constitutively express pathogen‐responsive genes and exhibit partial resistance to *Pseudomonas syringae* (Jung et al. 2020). These findings suggest that m^6^A‐mediated mRNA decay and UPF‐mediated NMD may act cooperatively to fine‐tune the abundance and quality of immune transcripts, ensuring timely activation and deactivation of defence responses.

The discovery of cross‐kingdom mRNA stability regulation has expanded our understanding of plant immune systems beyond canonical host‐pathogen interactions. Plant‐derived miRNAs encapsulated in extracellular vesicles are transported into insect vectors, where they differentially modulate transcript stability to balance viral propagation within insect bodies (Wang et al. 2025). Conversely, insect‐intrinsic small RNAs tune endogenous mRNA turnover to sustain pest fitness under microbial challenge (Guo et al. 2024). Plants employ mRNA decay to defend against pathogens and pests, which in turn evolve strategies to interfere with host mRNA decay. This dynamic interplay drives the coevolution between plants and biotic stressors. Despite notable advances across host‐virus defence, epitranscriptomic immune surveillance and cross‐kingdom RNA regulation, major unresolved gaps persist. The crosstalk linking mRNA decay machineries to epitranscriptomic marks and immune signalling remains poorly resolved, and field‐oriented investigations on combined‐stress scenarios are urgently required.

## 5 Research Value and Application Potential of mRNA Stability in Future Agriculture

Mounting evidence demonstrates that mRNA decay accurately shapes the spatiotemporal pattern of gene expression in plant growth and development, stress response and agronomic traits by dynamically regulating mRNA stability, endowing sessile plants with tunable environmental plasticity. Multilayered regulatory mechanisms, including NMD surveillance, m^6^A/ac^4^C modifications, LLPS and sRNA‐mediated transcript cleavage, are increasingly resolved, bringing strategies for manipulating RNA homeostasis from theoretical concepts closer to practical testing (Cai et al. 2025; Fan et al. 2024; Ma et al. 2025; Shen and Yu 2025; Tang et al. 2023; Yu, Zou, et al. 2024; Yu et al. 2021). A series of pioneering studies have demonstrated promising application prospects. For instance, a domestication‐related frameshift mutation in the cotton *GhGRDP1* gene regulates seed size via NMD‐mediated mRNA degradation, exemplifying how natural RNA‐metabolism variation contributes to crop domestication (Zhao et al. 2025). In field trials, expression of the mammalian m^6^A demethylase FTO reshaped mRNA stability and increased yields in rice and potato by approximately 50%, illustrating the substantial breeding potential of strategies that target RNA homeostasis (Yu et al. 2021). Furthermore, the CRISPR/Cas13 system enables precise and reversible degradation of endogenous mRNA, breaking through the limitations of traditional genetic manipulation and delivering a feasible experimental toolkit for targeted RNA‐metabolism perturbation (Yu, Zou, et al. 2024).

These strategies offer new avenues for directed breeding of high‐yielding, high‐quality, sustainable, and stress‐resilient crop varieties. Nevertheless, these promising laboratory and trial outputs require careful interpretation. Many early functional demonstrations rely on global perturbation of central mRNA decay effectors. Such broad intervention often triggers widespread transcriptome reprogramming and undesirable pleiotropic trade‐offs, which constrain direct adoption for real‐world breeding. This review systematically synthesizes recent advances in the mRNA decay regulatory network, with a focus on its multidimensional value and translational potential in future agriculture—including applicable scenarios, mechanistic insights, and technological breakthroughs, as well as translational pathways. It aims to efficiently empower intelligent breeding with this fine‐tuner of gene expression (Figure 5).

**FIGURE 5:**
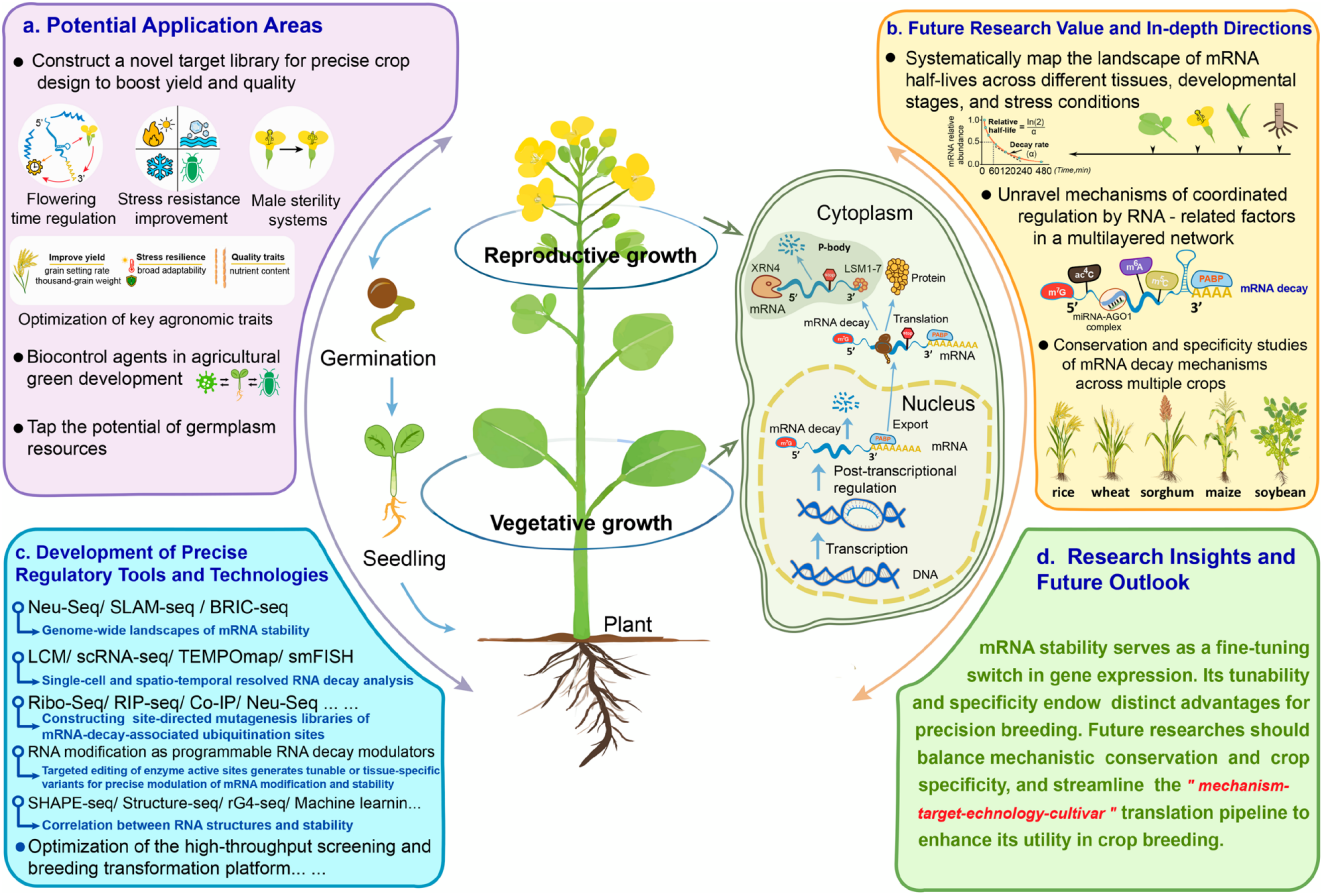
Research value and translational potential of mRNA stability regulation for crop improvement. (a) Agricultural application scenarios, key agricultural traits that can be improved via mRNA stability engineering. (b) Core research values and unresolved scientific questions, covering evolutionary adaptation, modular mechanism dissection, and cross‐kingdom regulatory dynamics. (c) Emerging precision tools for manipulating mRNA stability, such as base editing, epitranscriptomic modification, and targeted decay engineering. (d) Current research insights and future perspectives in this field.

### 5.1 Potential Agricultural Applications of mRNA Stability Regulation

In terms of potential application areas, based on the mechanism of mRNA stability regulation, a target library of mRNA stability for key agronomic traits can be systematically constructed (Figure 5a). For flowering‐time regulation, editing the m^6^A sites or uORF motifs of core genes such as *FT*, *SPL*, and *FLC* can finely tune their mRNA half‐life, thereby adapting the growth period to different photo‐thermal ecological regions (Nasim et al. 2021; Seo et al. 2011; Sureshkumar et al. 2016). For stress resistance improvement, targeted regulation of mRNA stability in stress‐responsive factors offers distinct advantages. For example, overexpressing *OsECT3* (deacetylation at K471 enhances *COLD1* mRNA stability) enhances cold tolerance (Ma et al. 2025). The development of male‐sterility systems can also benefit from this strategy. Either knocking out rice *OsALKBH9* or editing m^6^A sites of *TDR*/*GAMYB* can disturb pollen exine formation and generate thermo−/photo‐sensitive male‐sterile germplasm (Tang et al. 2024). More importantly, regulation of mRNA stability can directly improve yield and quality traits. For example, enhancing m^6^A modification of *PHR1* mRNA under phosphate deficiency activates the pathway for high‐efficiency phosphorus utilization (Liu, Wang, Wang, et al. 2025). Such a stability‐directed design strategy can avoid the metabolic burden or developmental imbalance caused by traditional overexpression, and is expected to enable safer, more precise, and tunable trait improvement. mRNA decay mechanisms also open new avenues for eco‐friendly crop protection. For instance, transgenically expressed peptides that block viral‐CP‐mediated Dcp1 ubiquitination restore host antiviral RNA‐degradation capacity (Wu et al. 2025). In addition, mRNA stability related pathways for pest control also hold great promise. dsRNA mimicking *Plutella xylostella* miR‐8545 can interfere with *PxGLD* mRNA stability, disrupt ecdysone homeostasis, and thereby enhance the insecticidal efficacy of Bt toxins (Guo et al. 2024). The biocontrol strategies based on RNA stability have the advantages of high target specificity, environmental friendliness, and low resistance risk, consistent with sustainable agriculture development.

Nevertheless, most of the above examples represent proof‐of‐concept observations. Direct wholesale perturbation of broad‐acting mRNA decay master regulators frequently triggers pleiotropic side effects and is seldom suitable for real world breeding. For instance, constitutive overexpression or knockout of m^6^A eraser coding gene *ALKBH10B* disturbs numerous downstream target transcripts and simultaneously interferes with flowering, developmental progression, and multiple stress response pathways. By contrast, natural allelic variation achieves fine‐tuned regulation without such costs: an enhanced native allele of *ALKBH10B* stabilizes *FT* transcripts to promote early flowering at high latitudes, whereas high temperature represses *FLM* through AS‐NMD to accelerate the reproductive transition in tropical environments (Duan et al. 2017; Sureshkumar et al. 2016). Drawing lessons from these natural variations, breeders should avoid manipulating the pleiotropic master regulator genes. Instead, we may edit m^6^A sites or uORF cis‐motifs within downstream floral‐regulatory genes (such as *FT*, *FLM*) to fine‐tune their mRNA half‐life and adapt crop life cycles to local photo‐thermal conditions. Therefore, translational work should prioritize three precision‐oriented routes: editing cis‐elements or RNA‐modification sites of trait‐associated transcripts, targeted modulation of key agronomic mRNAs, and rewiring reader‐target protein‐RNA interfaces.

### 5.2 Future Research and in‐Depth Directions for mRNA Stability in Crop Improvement

Given the promising application prospects of mRNA stability regulation, future research must be expanded in both mechanistic depth and functional breadth (Figure 5b). However, most current crop‐oriented observations remain largely correlative, and critical mechanistic gaps still disturb the translation of descriptive molecular observations into rational, trait‐oriented breeding design. Future research requires simultaneous advances to resolve unresolved regulatory puzzles and generate application‐ready knowledge for crop improvement. Firstly, we should systematically construct a transcriptome‐wide mRNA half‐life landscape of major crops across different tissues (root, stem, leaf, flower), developmental stages (germination, tillering, heading, grain filling), and abiotic stresses (drought, high temperature, salinity). This will reveal the principles governing mRNA stability and precisely identify key unstable transcripts that regulate yield and stress resistance, providing a foundation for target screening.

Then, it is necessary to go beyond the perspective of single modifications and systematically dissect the cooperative regulatory logic among multi‐layered factors, including m^6^A, ac^4^C, m^7^G, m^5^C, miRNAs, and ubiquitination. A set of conceptually critical yet poorly resolved questions demand dedicated experimental investigation, which directly shapes our capacity to implement precise post‐transcriptional crop breeding. (i) How do epitranscriptomic marks functionally cooperate with or antagonize RNA surveillance? Whether RNA modifications redefine NMD substrate selectivity, alter decay kinetics, or modify the recognition of PTC‐containing transcripts in crop tissues remains largely uncharacterized in crop systems. (ii) What constitutes the molecular interface between RNA‐modification‐governed mRNA decay and miRNA‐directed transcript cleavage? Whether shared substrate pools exist and how these two post‐transcriptional regulatory pathways mutually modulate target transcript fate represent outstanding open questions in plant mRNA control. (iii) Which core transcripts governing yield, fertility, and multi‐stress resilience are dually regulated by these interconnected post‐transcriptional modules? (iv) How do quantitative shifts in the abundance and translational output of these regulated transcripts propagate downstream and manifest as stable, heritable agronomic phenotypes under field conditions? For instance, we should clarify the crosstalk mechanisms between the m^6^A reader protein ECT8 and the decapping protein DCP5, as well as their interactions with LLPS and ABA signalling perception (Cai et al. 2024; Wu, Su, et al. 2024). These efforts will help establish a causal regulatory network of “RNA modification–binding protein–decay rate”.

Presently, most mRNA‐decay profiles are generated in *Arabidopsis*, and comparable multi‐condition, tissue‐resolved resources remain limited for specific crops, a major bottleneck in translating mechanistic insights from model plants into crop systems. Hence, given the potential divergence in regulatory conservation and specificity among crops, comparative functional studies should be conducted in parallel across major cereal and oil crops such as rice, wheat, maize, sorghum, and soybean. For example, it is necessary to evaluate the differences in regulatory strength and patterns of key pathways, including NMD in flowering genes, m^6^A in photosynthetic transcripts, and phasiRNA in anther's development. These analyses will provide a theoretical basis for intelligent breeding that balances universality and precision.

### 5.3 Technological Innovations for mRNA Stability Regulation in Crop Breeding

Translating mechanistic insights into mRNA stability control into practical crop breeding calls for targeted technical advances, rather than incremental application of existing multi‐omics workflows (Figure 5c). Current profiling pipelines often conflate transcriptional fluctuation with authentic mRNA turnover changes, hindering accurate identification of trait‐relevant unstable transcripts. To resolve this issue, the integration of nascent RNA labeling (Neu‐seq, SLAM‐seq), single‐cell transcriptomics, and spatially resolved techniques (LCM, smFISH, TEMPOmap) enables precise capture of dynamic, cell‐specific mRNA turnover patterns across developmental and stress contexts (Herzog et al. 2017; Ren et al. 2023; Szabo et al. 2020; Xue et al. 2025; Yu et al. 2025). Despite these technical advantages, high costs and demanding experimental workloads still restrict their large‐scale application in staple crops.

Beyond high‐resolution profiling, the development of programmable RNA stability regulators is essential to achieve precise trait manipulation. Direct perturbation of endogenous core mRNA decay factors usually triggers widespread transcriptome reprogramming and severe pleiotropic defects, thereby compromising plant growth and fitness. Instead of constitutive knockout or overexpression of master regulatory genes, future breeding efforts should prioritize engineered and modular RNA regulatory systems. Multidimensional screening can be performed by integrating Ribo‐seq, RIP‐seq, and Neu‐seq to precisely identify functional sites that regulate target mRNA stability (Guo et al. 2023; Xu et al. 2023; Yu, Zou, et al. 2024). Building on these efforts, programmable RNA stability regulatory tools can be further developed. CRISPR/Cas13 fused to decay effectors offers a programmable platform for locus‐specific degradation without altering genomic sequence (Cox et al. 2017; Shi et al. 2024; Wen et al. 2025). Such tools would allow breeders to fine‐tune gene expression dosage quantitatively rather than perform binary gene knockout.

Meanwhile, the integration of RNA structurome (SHAPE‐seq, rG4‐seq) and deep learning models (e.g., PlantRNA‐FM) enables quantitative prediction of how sequence variations affect mRNA secondary structure and half‐life, as well as intelligent inference of mRNA fate (Wu, Yu, et al. 2024; Yang et al. 2020; Yu, Yang, et al. 2024). Wu et al. demonstrated in wheat that domestication‐selected SNVs regulate mRNA stability by perturbing RNA structural motifs, validating the evolutionary and breeding significance of the *sequence–structure–stability* regulatory axis (Wu, Yu, et al. 2024). Ultimately, high‐throughput validation and translation platforms should be established, and efficient genetic transformation systems for staple crops such as rice and wheat should be optimized to complete the full chain from target discovery to variety breeding. Its programmability, spatiotemporal specificity, and environmental responsiveness confer unique advantages for precision crop design.

mRNA stability serves as a fine‐tuning regulator of gene expression. Its programmability, spatiotemporal specificity, and environmental responsiveness provide unique advantages for precision crop design. Future research should strike a balance between mechanistic conservation and crop specificity. It is necessary to explore core conserved regulatory modules across species to reduce research costs, while respecting the regulatory preferences of different crops to ensure practical application effects. As important, there is an urgent need to establish the full translational chain from mechanistic dissection, target identification, and tool development to variety breeding in future agriculture, and to build a standardized breeding technology system based on mRNA stability (Figure 5d). With continuous innovations in multi‐omics, artificial intelligence prediction, and gene‐editing tools, intelligent breeding based on mRNA stability is expected to become a revolutionary technology for safeguarding global food security and promoting green agricultural development.

## 6 Conclusions and Perspectives

Beyond simple transcript clearance, mRNA decay serves as an essential and evolutionarily ancient regulatory system sustaining eukaryotic gene expression homeostasis. The core components of mRNA decay pathways, including LSM1–7, CCR4–NOT, and UPF1–3 complexes, are universally conserved across eukaryotes. These conserved multi‐subunit machineries strongly support the LECA complexity hypothesis by supplementing evolutionary evidence from the endomembrane system, cytoskeleton, and cell cycle machinery, and also represent robust phylogenetic markers that improve the resolution and reliability of eukaryotic evolutionary reconstruction. Besides, plant mRNA decay research has progressed from individual gene characterization to genome‐wide network profiling, revealing its critical roles in regulating plant development, stress responses, and key agronomic traits.

Despite these advances, the field faces notable limitations restricting evolutionary interpretation and agricultural translation. Current functional validation is heavily biased toward *Arabidopsis*, with insufficient evidence from crops and other non‐model plants, impeding the establishment of universal cross‐species regulatory rules. Evolutionarily, strong purifying selection stabilises the core decay scaffold with lethal mutations rarely tolerated, whereas auxiliary regulatory elements and RNA target sites exhibit high evolutionary plasticity, acting as key drivers of plant adaptive evolution. However, the modular boundaries between conserved core machinery and editable regulatory regions remain ambiguous, and their divergent contributions to environmental adaptation are poorly resolved. Furthermore, the targeted selection patterns of decay genes during crop domestication, as well as the synergistic regulation of multiple decay pathways under complex combined stresses, are still largely uncharacterised.

From the translational perspective, conventional global mRNA modulation strategies, despite their promising yield‐improving potential, frequently cause off‐target effects and developmental imbalance, limiting their practical agricultural application. Future studies should integrate multi‐omics analysis, functional verification, and agronomic phenotypic evaluation to construct crop‐specific mRNA stability regulatory networks and comprehensive evolution–function landscapes of eukaryotic decay complexes. Such work will deepen our understanding of eukaryotic origin and adaptive diversification, while excavating precise crop breeding targets. Moreover, the development of spatiotemporally precise editing tools will enable accurate dissection of cross‐kingdom mRNA decay dynamics between plants and environmental organisms, ultimately translating basic mechanistic findings into safe, efficient, and sustainable crop improvement strategies.

## Author Contributions

Yuchi He, Haidan Wu and Yelin Huang conceived and designed the study. Haidan Wu and Dayin Lv participated in data collection. H.W. analysed the data and drafted the manuscript. H.W. prepared the model figures. Y. He., H.W., Y. Hu. and D.L. revised the manuscript. All authors read and approved the final version of the manuscript.

## Funding

This work was supported by the China Postdoctoral Science Foundation (2025M774018), Guangdong Provincial Key Laboratory of Plant Stress Biology Open Project (2025PlantKF03).

## Conflicts of Interest

The authors declare no conflicts of interest.

## Data Availability

The data that support the findings of this study are available in Phytozome at https://phytozome‐next.jgi.doe.gov/. These data were derived from the following resources available in the public domain: UCSC Genome Browser, http://genome.ucsc.edu/.
